# Hyperbranched Polyelectrolyte Copolymers as Novel Candidate Delivery Systems for Bio-Relevant Compounds

**DOI:** 10.3390/ma16031045

**Published:** 2023-01-24

**Authors:** Anastasia Balafouti, Stergios Pispas

**Affiliations:** Theoretical and Physical Chemistry Institute, National Hellenic Research Foundation, 48 Vassileos Constantinou Ave., 11635 Athens, Greece

**Keywords:** amphiphilic copolymers, hyperbranched copolymers, polyelectrolytes, RAFT polymerization, self-assembly, curcumin encapsulation, lysozyme complexation

## Abstract

In this study, reversible addition-fragmentation chain transfer (RAFT) polymerization is utilized in order to synthesize novel hyperbranched poly(oligoethylene glycol) methyl ether methacrylate-co-tert-butyl methacrylate-co-methacrylic acid) (H-[P(OEGMA-co-tBMA-co-MAA)]) copolymers in combination with selective hydrolysis reactions. The copolymers showing amphiphilicity induced by the polar OEGMA and hydrophobic tBMA monomeric units, and polyelectrolyte character due to MAA units, combined with unique macromolecular architecture were characterized by physicochemical techniques, such as size exclusion chromatography (SEC) and ^1^H-NMR spectroscopy. The hyperbranched copolymers were investigated in terms of their ability to self-assemble into nanostructures when dissolved in aqueous media. Dynamic light scattering and fluorescence spectroscopy revealed multimolecular aggregates of nanoscale dimensions with low critical aggregation concentration, the size and mass of which depend on copolymer composition and solution conditions, whereas zeta potential measurements indicated pH sensitive features. In addition, aiming to evaluate their potential use as nanocarriers, the copolymers were studied in terms of their drug encapsulation and protein complexation ability utilizing curcumin and lysozyme, as a model hydrophobic drug and a model cationic protein, respectively.

## 1. Introduction

Polymer-based materials have recently become a vast research area in the field of therapeutics. From implants and medical devices to medicinal delivery platforms, the synthetic polymer industry has enhanced modern health treatment with several clinically translated and developing technologies [1]. Among these technologies, polymer nanoparticles (PNPs) have almost monopolized the research as an alternative and more beneficial administrating route of bioactive compounds into the human body [2]. Poor solubility in aqueous media, inherent toxicity, and low bioavailability are common limitations for many active pharmaceutical or diagnostic agents [3]. PNP development driven by amphiphilic copolymer self-assembly has been established as a promising method to surpass these obstacles. Upon dissolution in aqueous media, amphiphilic copolymers tend to assemble into core-shell-like structures composed of hydrophobic segments, able to serve as an exclusive host to poorly soluble compounds, surrounded by hydrophilic segments competent to shield the referenced structure and confer stability in physiological media [4]. The core-shell-like PNPs usually display nanoscale dimensions of 1–100 nm [5], whereas a wide range of these were mentioned in reviews by several groups for presenting properties such as high drug loading capacity, the ability of specific target drug release, high circulation time and stability, as well as biodegradability [6,7,8]. Due to the recent progress in polymerization techniques and especially in reversible-deactivation radical polymerizations (RDRP) including nitroxide-mediated polymerization (NMP), atom transfer radical polymerization (ATRP), and reversible addition-fragmentation chain transfer (RAFT) polymerization, it is possible to obtain a plethora of copolymer compositions and architectures, with characteristic flexibility in customized properties [9]. The macromolecular architecture of amphiphilic copolymers influences their self-assembly behavior to a great extent [10,11,12].

Hyperbranched copolymers (HCs) are a class of macromolecules with unique three-dimensional structures composed of randomly regulated branching units. Amphiphilic copolymers with hyperbranched architecture are lately more excessively investigated in terms of their self-assembly behavior since, in many cases, they are proven to present better qualities than their linear analogs in respect of delivery applications [13,14,15]. For example, Bera and coworkers reported a comparative study between linear and hyperbranched amphiphilic poly(disulfides), where hyperbranched polymeric assemblies presented much higher loading content and efficiency for the intracellular delivery of doxorubicin drug [16]. Similarly, Tang and colleagues described the beneficial role of hyperbranched polymers of palmitic acid-modified polyethylenimines in contrast to linear architecture in an attempt to coat and stabilize citrate-protected 17-nm gold NPs [17]. In addition, Yan and his team described how amphiphilic hyperbranched copolymers with polyphosphate arms were able to self-assemble into spherical micelles, which exhibited excellent biocompatibility and efficient cellular uptake in an in vitro evaluation [18]. Apart from high encapsulation efficiency and enhanced stability, another commonly reported advantage related to hyperbranched polymer architecture is the high density of functional groups at the periphery of the globular structure. Last but not least, HCs benefit from facile synthetic routes generally carried out in a one-pot reaction [19]. A very practical route to obtain hyperbranched copolymers is utilizing an RDRP method in combination with a multifunctional monomer for the establishment of branching points [13,20,21]. 

In the present report, we describe the synthesis of novel hyperbranched poly(oligo ethylene glycol methyl ether methacrylate-co-tert-butyl methacrylate-co-methacrylic acid), P(OEGMA-co-tBMA-co-MAA) copolymers, the design of which aims at the development of self-assembled PNP systems intended to serve as potential nanocarriers for bio-relevant compounds. RAFT polymerization in combination with multifunctional comonomer ethylene glycol dimethacrylate (EGDMA) as a branching agent was utilized to obtain P(OEGMA-co-tBMA) precursor copolymers, while a post-polymerization hydrolysis reaction was carried out to chemically modify a portion of the tBMA units into MAA, so as to induce polyelectrolyte character. OEGMA and tBMA-based polymers are biocompatible macromolecules presenting intense hydrophilic and hydrophobic character, respectively, whereas OEGMA provides stealth-like properties [22,23]. Poly(methacrylic acid) (PMAA) is a biocompatible weak anionic polyelectrolyte normally constituting a functional component of stimuli-responsive copolymers. Weak polyelectrolytes partially dissociate when dissolved in an aqueous medium and, as a consequence their total charge, changes as a function of pH. This property enables complexation with oppositely charged components, while in certain circumstances, the ability of targeted drug release is demonstrated [24,25,26,27]. 

Herein the P(OEGMA-co-tBMA-co-MAA) HCs bearing amphiphilicity and polyelectrolyte character combined with unique macromolecular architecture are explored further and analyzed towards their self-assembly behavior in aqueous media, and finally, their potential as biomaterials is evaluated by studying their protein complexation and drug incorporation ability while utilizing model biocompounds lysozyme (Lys) and curcumin (CUR), respectively. 

## 2. Materials and Methods

### 2.1. Materials 

Monomers tert-butyl methacrylate (tBMA), oligo ethylene glycol methyl ether methacrylate (OEGMA) (average M_n_ = 950 g/mol) (dissolved in 1,4-dioxane) and ethylene glycol dimethacrylate (EGDMA) were passed through a column packed with inhibitor removers (butylated hydroxytoluene and hydroquinone monomethyl ether, from Sigma-Aldrich, Athens, Greece) for purification. We recrystallized 2,2′-azobisisobutyronitrile (AIBN) from methanol, and 1,4-dioxane (99.8% pure) was dried over molecular sieves. N-hexane (96% pure), tetrahydrofuran (THF), trifluoroacetic acid (TFA), deuterated chloroform (CDCL_3_), 4-cyano-4-(phenyl-carbonothioylthio) pentanoic acid (CPAD), pyrene, curcumin (CUR), and lysozyme (Lys) were used as received. Materials and chemicals mentioned above were purchased from Sigma-Aldrich, except CUR and EGDMA which were purchased from Merck.

### 2.2. Synthesis of H-[P(OEGMA-co-tBMA-co-MAA) Copolymers

#### 2.2.1. Preparation of H-[P(OEGMA-co-tBMA)] Copolymers via RAFT Polymerization

Two hyperbranched P(OEGMA-co-tBMA) copolymers with differing hydrophilic OEGMA and hydrophobic tBMA monomer compositions were synthesized by reversible addition-fragmentation chain transfer (RAFT) polymerization in the presence of a divinyl monomer, EGDMA as a branching agent. AIBN and CPAD were used as the radical initiator and chain transfer agent, respectively, whereas the selected solvent for the reaction was 1,4-dioxane. The general procedure, including detailed information for HC 1 copolymer synthesis, is described in the following. OEGMA (1.4 g, mmol), tBMA (0.6 g, mmol), EGDMA (0.076 mL, 0.4 mmol), AIBN (16.421 mg, 0.1 mmol), CPAD (55.876 mg, 0.2 mmol), and 1,4-dioxane (10 mL of total solution) were placed in a 25 mL round bottom flask sealed with a rubber septum, and a magnetic stirring bar was added. The reaction mixture was first deoxygenated for 15 min by nitrogen bubbling and afterward left at 70 °C in an oil bath while stirring. After 24 h of the polymerization reaction, the solution was directly placed at −20 °C for approximately 20 min and then exposed to air for termination of the polymerization. The polymer solution was preventively dialyzed through a 3.5 kDa MWCO membrane against deionized water for the removal of any low molecular weight oligomers or unreacted monomers. A rotary evaporator was used in order to concentrate the dialyzed solution, which was next dried in a vacuum oven at room temperature for 48 h and finally collected and stored in a refrigerator. Size exclusion chromatography (SEC), ^1^H nuclear magnetic resonance (^1^H-NMR) spectroscopy, and dynamic light scattering were used to molecularly characterize the copolymers. Collective molecular characterization results are shown in Table 1.

#### 2.2.2. Preparation of H-[P(OEGMA-co-tBMA-co-MAA)] Copolymers via Hydrolysis Reaction 

The hyperbranched P(OEGMA-co-tBMA) copolymers were hydrolyzed using TFA in order to obtain P(OEGMA-co-tBMA-co-MAA) copolymers. A typical procedure with detailed information for HC 1 is described as follows: A 30 mL solution of P(OEGMA-co-tBMA) (0.6 g) in THF was placed in a round bottom flask with a magnetic stirring bar. TFA (0.485 mL) (5 times molar excess to tBMA units) was then added to the mixture, and the flask was sealed and covered with aluminum foil for protection against light. In general, a five times equivalent excess of TFA to tBMA was used. After about one week of hydrolysis reaction at room temperature, the solution was concentrated using a rotary evaporator, while the resulting product was dried in a vacuum oven for 48 h in order for the solid residue to be collected and stored in the refrigerator. ^1^H-NMR and Fourier–transform infrared spectroscopy were utilized to evaluate the hydrolysis reaction yield.

### 2.3. Preparation of Self-Assembled PNPs in Aqueous Solutions

Considering the copolymer’s hydrophobicity, we used the organic solvent displacement protocol [28] to prepare P(OEGMA-co-tBMA-co-MAA) nano-aggregates in aqueous media at concentrations of 5 × 10^−4^ g/mL. Specifically, the dry solid copolymers were initially dissolved in a small quantity of THF. The copolymer solutions were then injected, in a rapid manner, into the proper amount of distilled water under vigorous stirring. After approximately one minute of stirring, the mixture was heated so that the organic solvent was evaporated.

### 2.4. Preparation of HC–Lys Complexes

Solutions of HC 2 and Lys in distilled water were separately prepared. The HC solutions were once again prepared via the organic solvent displacement protocol. Three sample series for complexation studies were prepared, where each series included two or five charge ratios of HC (negatively charged component) and Lys (positively charged component). For each series, the concentration of the HC was kept constant for all different ratio samples, whereas the Lys concentration varied. Particularly, three solutions of HC of different concentrations were prepared, and then each was equally divided into two or five portions. The appropriate amount of Lys solution according to the desired charge ratio was then added to the polymer solution by a rapid injection under stirring. 

### 2.5. Preparation of CUR Loaded Aggregates 

A similar protocol was used for the investigation of drug encapsulation ability. Samples of both HCs with 20% weight of CUR to copolymer weight were prepared. The proper amount of CUR and copolymer were first separately dissolved in THF, and the CUR-THF mixture was added to the THF-copolymer mixture. The resulting solutions were then added rapidly by injection in distilled water under vigorous stirring. After a while, the solutions were heated until the organic solvent evaporated.

### 2.6. Experimental Techniques 

#### 2.6.1. Size Exclusion Chromatography 

A Waters Corporation size exclusion chromatography set equipped with a Waters 1515 isocratic pump, a set of three μ-Styragel mixed pore separation columns of pore diameter ranging from 102 to 106 Å, and a Waters 2414 refractive index detector equilibrated at 40 °C were used. THF (with 5% *v*/*v* triethylamine) was utilized as the eluent at a flow rate of 1.0 mL/min. Calibration of the column set was performed with linear monodisperse polystyrene standards of average molecular weights between 1200 and 152,000 g mol^−1^. Breeze software was used for data acquisition and analysis.

#### 2.6.2. Proton Nuclear Magnetic Resonance Spectroscopy (^1^H-NMR)

^1^H NMR measurements of HC samples were performed on a Varian 300 (300 MHz) spectrometer with Vjnmr Software. CDCl_3_ was used as the solvent for sample preparation (c ≈ 14 mg/mL). Chemical shifts are given in parts per million (ppm) using tetramethylsilane as an internal reference, and the results were analyzed by MestReNova Software.

#### 2.6.3. Attenuated Total Reflectance-Fourier Transform Infrared (ATR–FTIR) Spectroscopy

FTIR spectra of dry solid copolymer samples were recorded on a Bruker (Billerica, MA, USA) Equinox 55 Fourier transform spectrometer, equipped with a single bounce ATR diamond accessory (Dura-Samp1IR II by SensIR Technologies, Danbury, CT, USA). Every spectrum was received as the average of 64 scans collected in the 5000 to 500 cm^−1^ spectral range and at 4 cm^−1^ resolution.

#### 2.6.4. Dynamic Light Scattering (DLS)

DLS studies were carried out with an ALV/GS-3 compact goniometer system (ALV GmbH, Hessen, Germany) with a JDS Uniphase 22 mW He–Ne laser, operating at 632.8 nm wavelength. The system is equipped with an ALV/LSE-5003 light scattering electronics unit used for stepper motor drive and limit switch control and an ALV-5000/EPP multi-τ correlator including 288 channels. The obtained correlation functions (and the simultaneously recorded light scattering intensity) were the average of five measurements at a goniometer angle of 90°, analyzed by the cumulants method and the CONTIN algorithm. All aqueous and organic solutions were filtered through 0.45 μm hydrophilic PVDF filters and hydrophobic PTFE filters, respectively. Samples studied were of HC concentration (C_HC_) = 5 × 10^−4^ g/mL, unless stated otherwise.

#### 2.6.5. Electrophoretic Light Scattering (ELS)

Ζeta potential values (related to the surface charge) were measured by electrophoretic light scattering experiments conducted on a Nano Zeta Sizer instrument from Malvern which is equipped with a 4 mW He–Ne, operating at 633 nm and a scattering angle of 173°. Each measurement was the average of approximately 25 repeated scans and the obtained data were analyzed by the Smoluchowski equation.

#### 2.6.6. Fluorescence Spectroscopy

Fluorescence measurements were conducted on a Fluorolog-3 Jobin Yvon-Spex spectrofluorometer (model GL3-21). For pyrene fluorescence measurements, the emission spectra were collected in the range 355–630 nm, and the excitation wavelength used was λ_ex_ = 335 nm. Sample preparation for the copolymer solutions involved a successive dilution of a stock solution resulting in 11 copolymer solutions in the concentration range of 5 × 10^−4^ to 5 × 10^−9^ g/mL. The highest concentration (stock) solution was prepared following the aforementioned organic solvent displacement protocol. Each solution was mixed with the proper amount (1 μL/mL) of 1 mM pyrene solution in acetone and was then left overnight at room temperature in the dark for the acetone to evaporate. With respect to Lys (tryptophan is the fluorescence active residue) and CUR spectra, the emission spectrum was collected in the range of 310–550 and 425–790 nm, while the excitation wavelength used was λ_ex_ = 290 and 405 nm, respectively. For CUR loading studies, 0.5 mL of the corresponding sample studied by DLS was diluted in 2.5 mL of water, whereas samples of Lys complexes were measured as such. 

#### 2.6.7. UV-Vis Spectroscopy

UV-Vis spectra were recorded on a Perkin Elmer Lambda 19 spectrometer in the range of 200–800 nm. CUR-loaded PNPs formed by HCs were investigated in terms of loading capacity and encapsulation efficiency. For each measurement, 0.5 mL of the corresponding sample studied by DLS was diluted in 2.5 mL of water or acetone (C_HC_ = 1.67 × 10^−4^ g/mL).

## 3. Results and Discussion

### 3.1. HC Synthesis and Characterization

**Scheme 1:**
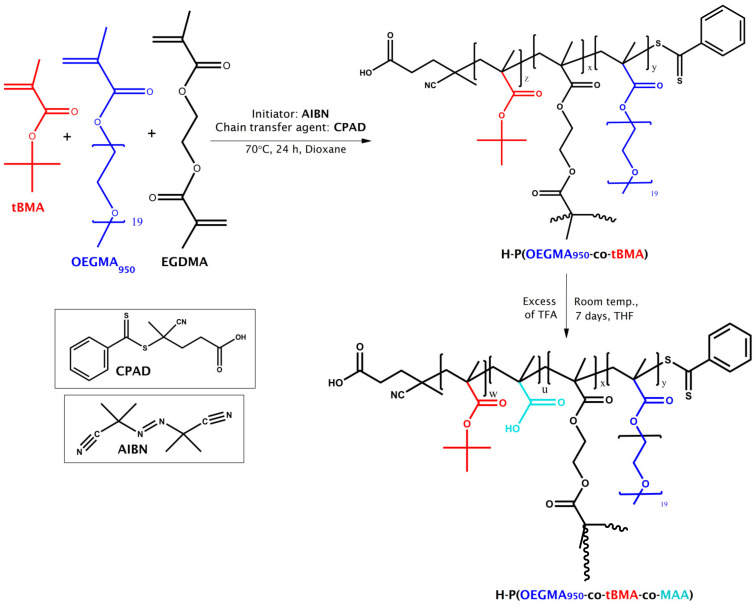
Molecular characteristics of the synthesized HCs.

**Figure 1:**
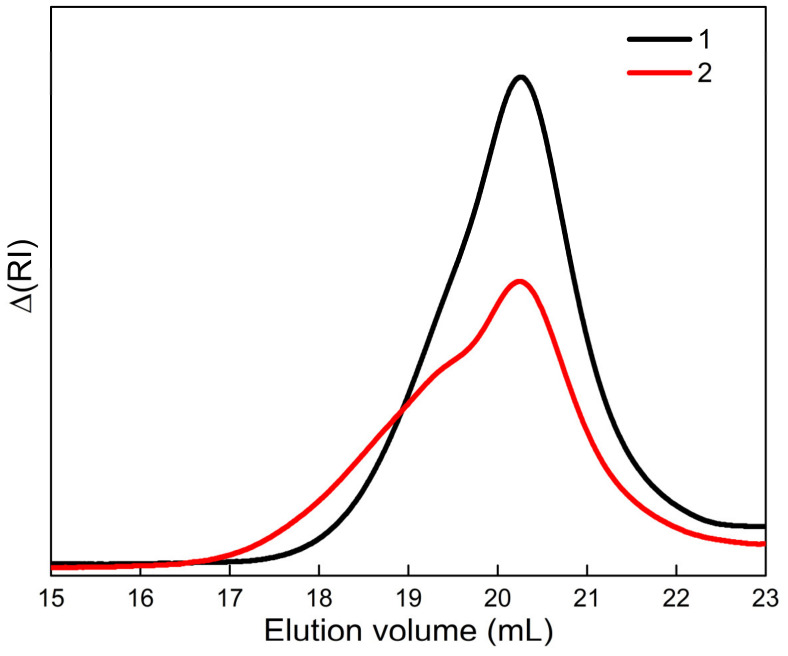
SEC traces of the precursor HCs (before hydrolysis) synthesized by RAFT.

High conversion hyperbranched P(OEGMA-co-tBMA) copolymers with different composition were successfully prepared via the one step process of RAFT copolymerization of OEGMA, tBMA, and EGDMA, as illustrated in Scheme 1. The molecular characteristics determined are presented in Table 1. The inclusion of EGDMA monomer in a small ratio compared to chain transfer agent CPAD is responsible for the final hyperbranched architecture. EGDMA is a common difunctional methacrylic monomer that contains two vinyl groups. The two vinyl groups are therefore of equal reactivity; however, when a vinyl group on one side is attached to a growing polymer chain, the steric environment and the reactivity of the other vinyl group, which is now pendant on the growing chain, suddenly alters [29]. Both intermolecular and intramolecular crosslinking are possible under these conditions. According to the literature, controlled limitation of intermolecular crosslinking, cyclization reactions, knotting, or networking is feasible by managing the monomers and CTA to EGDMA ratio [30,31]. Size exclusion chromatograms of the copolymers are displayed in Figure 1. A mild shoulder is observed at the left side of the main peak of the HC 2 chromatogram, which is probably attributed to higher molecular weight cycled or knotted chains formed by the aforementioned side reactions. Nevertheless, the chromatograms depict monomodal molecular weight distributions with polydispersity indexes around 1.2, which is a value within the range imposed by the theoretical background and the common practical application of the RAFT polymerization technique for hyperbranched copolymers [32,33,34]. The absolute values of weight average molecular weights are much higher than the estimated values by SEC since SEC calibration was performed with linear PS standards.

**Figure 2:**
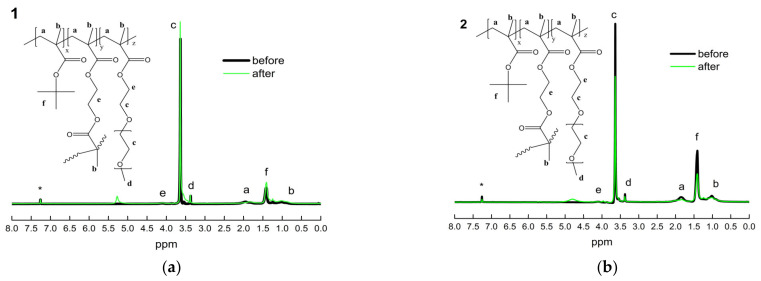
^1^H-NMR spectra of the HC 1 (**a**) and HC 2 (**b**) in CDCl_3_ before and after hydrolysis.

The percent ratio between the incorporated monomers in the copolymers was determined by ^1^H-NMR measurements. ^1^H-NMR spectra and peak assignments are depicted in Figure 2. Based on the integrated intensities of the signal at δ 3.64 ppm, corresponding to the repeating methylene protons of OEGMA pendant chains [35], and the signal at δ 1.41 corresponding to the methyl protons in the tert-butyl groups of tBMA [27], the HCs chemical compositions are in complete accordance with the initial monomer feed compositions (the asterisk in the relative peak presented in Figure denotes the solvent signal).

Since molecular characterization revealed the success of the polymerization reaction, polymer samples were hydrolyzed to obtain the polyelectrolyte form of the copolymers containing MAA units. The general hydrolysis reaction procedure is also presented in Scheme 1. The final HCs compositions were likewise obtained by calculating the, now diminished, integration intensity of the characteristic peak of the tert-butyl group in ^1^H-NMR spectra before and after hydrolysis towards OEGMA peaks, which remain unaffected by hydrolysis (Figure 2). FT-IR spectroscopy measurements of the solid HCs were also used for a qualitative comparison. 

**Figure 3:**
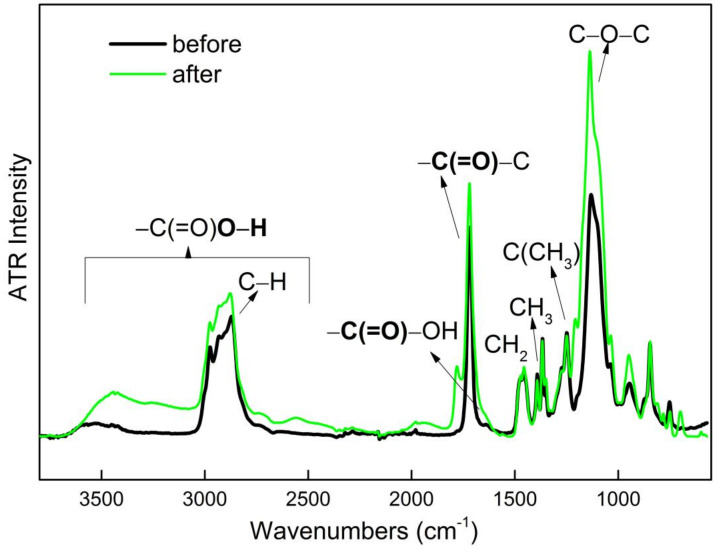
FT-IR spectra of HC 2 before and after hydrolysis.

Figure 3 shows the FT-IR spectra of HC 2 solid samples before and after hydrolysis that confirm the chemical structure variation. The spectra were normalized to the characteristic band of –CH_2_-bending vibrations (medium peak at ca. 1477 and 1455 cm^−1^) considered an unaltered segment after hydrolysis. The absorption bands associated with the tBMA segments (1392, 1365, and 1249 attributed to –CH_3_- bending vibrations and –C(CH_3_)_3_ skeletal vibrations, respectively) are present with analogous intensity in both spectra, yet a characteristic broad band between 2500 and 3550 cm^−1^ assigned to stretching vibrations of carboxylic acid hydroxyls –C(=O)**O–H** appears after hydrolysis. Moreover, a slight shift of the ester carbonyl stretching vibration band near 1720 cm^−1^ to a broader and lower wavelength number indicates the conversion of some ester groups to carboxylic acid groups, whereas after hydrolysis, the peak is also slightly split, a phenomenon previously reported as a sign of hydrogen bonding between methacrylic acid and ethylene glycol units. Lastly, the intensity of the absorption peaks related to the ester groups (near 1720 and 1135 cm^−1^, assigned to the stretching vibrations of –**C(=O)**–O–C and –**C**(=O)**–O**–, respectively) is not affected, indicating that the backbone of the polymer chain is not damaged [36,37,38,39]. FT-IR spectra of solid HC 1 before and after hydrolysis are included in Figure S1 of the Supplementary Materials.

Even though the reaction conditions were selective for complete hydrolysis of the tertiary butyl groups, a rather low conversion was observed for both copolymers (Table 1). Considering that branched PtBMA precursor was reported to completely convert to PMAA under similar conditions [40], a possible explanation for this result is that the high excluded volume OEGMA oligoethylene pendant chains attached to the hyperbranched macromolecular chain spatially affect the hydrolysis reactionthrough steric hindrance. This hypothesis is also supported by the fact that HBC 2 with 20% higher tBMA content than HBC 1 displayed 66% higher hydrolysis yield, as well as by the observed behavior of the copolymers when dissolved in THF. 

THF is a common solvent for all three OEGMA, tBMA, and MAA units, thus the HCs exist in a state close to molecularly dissolved chains. However, MAA is less soluble compared to tBMA, since its miscibility with the particular organic solvent is based on the formation of specific hydrogen bonds [41]. DLS measurements (see Supplementary Materials Figure S2 and Table S1) of the HCs in this solution state revealed that the average hydrodynamic radii (R_h_) are moderately larger after hydrolysis, probably reflecting partial aggregation of some chains indicating that due to steric hindrance, the copolymers are mainly surface and less internally modified.

### 3.2. Self-Assembly Studies of Amphiphilic Polyelectrolyte Hyperbranched Copolymers in Aqueous Media

#### 3.2.1. CAC determination

**Figure 4:**
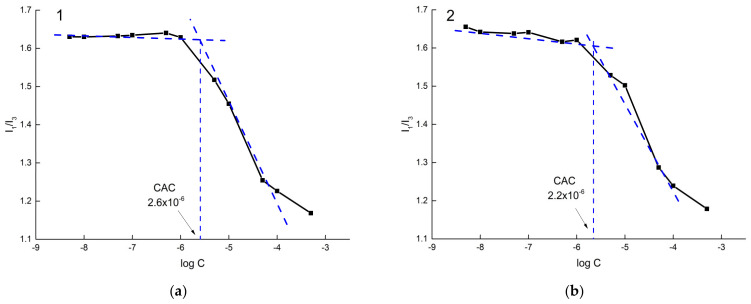
CAC determination for HC 1 (**a**) and HC 2 (**b**) amphiphilic polyelectrolyte copolymers in water.

The hydrolyzed HCs now bearing both amphiphilic and polyelectrolyte character were subsequently studied towards their self-assembly behavior in aqueous solutions. A fluorescence assay utilizing pyrene as a probe was employed to determine the HCs critical aggregation concentration (CAC). CAC, meaning the lowest concentration in which the HCs form core-shell particles at the nanoscale, is a declaratory parameter for polymeric nano-systems designed for bio-applications. Low CAC values are desirable, since potential dissociation from a relative core-shell form upon immense dilution in physiological media, that is, once placed in an in vivo environment, diminishes the system stability and biofunctionality. Pyrene is a fluorescent hydrophobic probe with characteristic emission spectra distinctive of the micro-environment polarity around it. Figure 4 shows the variation of the intensity of the first peak (I_1_ around 371 nm) to the third peak (I_3_ around 382 nm) of pyrene’s emission spectra when mixed with the HCs, as a function of the logarithm of HCs concentration. 

High I_1_/I_3_ values represent polar hydrophilic environment, whereas low I_1_/I_3_ values indicate that pyrene is localized in the non-polar hydrophobic environment of the HCs core domain. The intersection between the two tangent lines to the curves data displaying a sharp decrease in the I_1_/I_3_ value is defined as the CAC [42]. The two HCs possess similar hydrophobic content, however, HC 1 macromolecules include higher hydrophilic mass due to OEGMA pendant chains causing a different balance in amphiphilicity, and therefore in self-assembly behavior. As expected, HC 2, which is more hydrophobic, presents a slightly lower CAC value [43]. These low CAC values suggest the thermodynamic stability of the formed aggregates and denote the presence of hydrophobic domains within them [44].

#### 3.2.2. Physicochemical Characterization of Self-Assembled Aggregates

**Figure 5:**
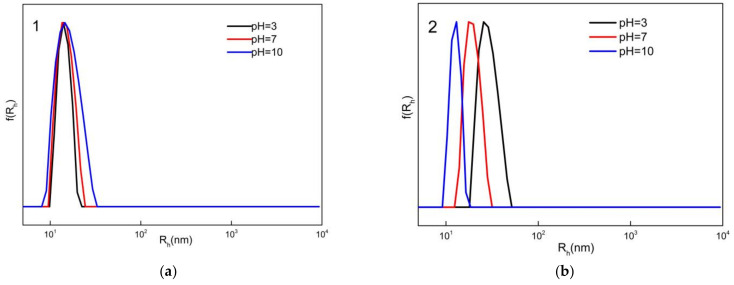
Size distributions from DLS analysis of HC 1 (**a**) and HC 2 (**b**) self-assembly in aqueous media at different pH values.

Next, a dynamic light scattering study of aqueous solutions of the HCs at concentrations above the CAC, where they are already known to form aggregates, was carried out to receive information about the apparent hydrodynamic radii (R_h_) and the size polydispersity indices (PDI) of the nanostructures formed. Considering that MAA charge content is pH-dependent, copolymer solutions at pH 3, 7, and 10 were prepared to examine whether the HCs self-assembly is affected by pH. Figure 5 shows size distributions from CONTIN analysis for each HC. It is noted that both HCs form monomodal distributions of small-size aggregates in water. Since the measured R_h_ of the aggregates is larger than the measured R_h_ of the HCs as individual macromolecular chains in THF, it is safe to state that the nanoparticles observed in water consist of multi-molecular aggregates. This is corroborated by the substantial increase of scattered light intensity (I) in water compared to THF solutions (Table 2 and Table S1). For example, regarding HC 1, the scattered intensity in the THF solution of C_HC_ = 10^−2^ g/mL was at least 10 times lower than the measured intensity in the aqueous solution, which incidentally was of a lower concentration of the HC copolymer. In addition, the low MAA content of HC 1 seems to have a negligible effect on the size variation in response to pH changes, whereas more noticeable pH-dependent self-assembly is observed for HC 2. Specifically, in contrast to what is usually observed for amphiphilic copolymers containing MAA units, the size of the PNPs decreases when the pH value increases. 

Normally at high pH values, over the apparent pKa of MAA (~4.8), micellar NPs of MAA copolymers display swelling of the corona due to MAA deprotonation, although this pH-size dependence is not always linear [39,45]. In the present case, at pH 7, where partial ionization is expected to occur, an average R_h_ of 19 nm is observed, and at pH 10 with expected complete ionization, an average R_h_ of 13 nm is observed. This slight size decrease may be correlated with the readjustment of the hyperbranched chains into a higher number of smaller multi-molecular aggregates. The comparison of the measured scattered light intensity between the two pH conditions is also consistent with this assumption since at pH 10, scattered light intensity is 10 times lower than at pH 7, evincing that the aggregates consist of significantly less mass. Accordingly, at pH 3, DLS measurements revealed aggregates with an average R_h_ of 29 nm and a respective scattered light intensity reduced by half when compared to the intensity at pH 7. Hence, larger and looser structures are formed, while notably, loss of ionization does not cause the collapse of the system. Since PMAA units become less hydrophilic but not completely hydrophobic in acidic conditions, this is probably attributed to the fusion or inclusion of more available polymer chains in the aggregates, for restoration of the hydrophilic–hydrophobic balance of the aggregates. Considering all the above states of copolymer chains, it is important to note that electrostatic repulsions due to ionization of the MAA functional groups are also affected by the copolymer macromolecular architecture. The random, or similarly statistical, distribution of MAA units along the polymer hyperbranched structure carrying a higher number of tBMA and OEGMA units may undermine electrostatic repulsions or even constitute an antagonizing factor to macromolecular swelling.

Furthermore, zeta potential (ζ_p_) measurements conducted by electrophoretic light scattering confirm the increase of ionization with the increase of pH.

### 3.3. Protein Complexation Studies

**Figure 6:**
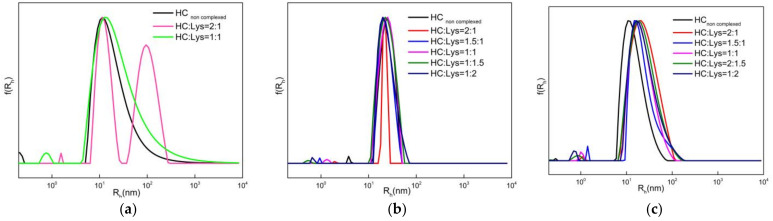
Size distributions of the HC–Lys complexes at HC concentrations of (**a**) 0.43 × 10^−4^, (**b**) 0.75 × 10^−4^ and (**c**) 1.87 × 10^−4^ g/mL.

Since HC 2 presents self-assembled nanoparticulate aggregates bearing negative surface charge in aqueous media, egg white lysozyme (Lys) was utilized in order to investigate the copolymers protein complexation ability through electrostatic interactions. Lys is a model cationic, water-soluble, relatively stable globular protein, with a total positive charge of +8 per molecule at pH conditions below its isoelectric point [46]. It is known for its antibacterial function due to cell lytic activity and thereby usually selected for drug delivery studies [47,48,49]. Complexation was studied for three different HC concentrations (a) 0.43 × 10^−4^, (b) 0.75 × 10^−4^, and (c) 1.87 × 10^−4^ g/mL at pH 7, while several negative-to-positive charge mole ratios between HC 2 and Lys were examined for each copolymer concentration. Ratios that include the HC at concentration (a) will be referred to as series (a), ratios at concentration (b) will be referred to as series (b,) and at concentration (c) as series (c), respectively, for brevity. Table 3 displays the results from DLS and ELS analysis of all copolymer/Lys complexes for series (a), (b), and (c), whereas size distributions from CONTIN analysis are shown in Figure 6.

In all cases, comparison of size distributions and zeta potential measurements of samples before and after adding Lys indicates that complexation via electrostatic interactions is taking place. The shift of size distributions and scattered light intensity towards higher values besides the shift of zeta potential from negative to positive near neutral values are the general trends that lead to this conclusion. This trend, however, is not dramatic, since it can be observed from comparing results from different HC:Lys ratios that increasing the concentration of Lys does not necessarily cause the emergence of complexes with much greater size. Interestingly, in the case of series b and c complexes, the increase of average R_h_ of the complexes is somehow small if compared to the increase in the scattered light intensity. Considering this and the fact that no other aggregate population appears, except for a very small percentage (<3% in every case) of molecules of R_h_ around 0.5–2 nm attributed, most probably, to a few non-complexed Lys molecules, it can be assumed that Lys is attracted and distributed on the surface of the already formed aggregates in a decorative manner. Additionally, as expected, these phenomena are more intense at higher concentrations and therefore easier to observe (case (c)). 

After all, self-assembly behavior variations of the HC in each concentration affect the resultant complexes. At lower concentrations of HC 2 (case a) an HC:Lys = 2:1 ratio induces the formation of complexes of two-size populations with large deviation, which could be a result of the low size homogeneity (PDI > 0.4) of the aggregates before adding Lys. In addition, it is possible that Lys complexation dissociates some copolymer aggregates and some Lys molecules form bridges between one or more HC aggregates, therefore some NPs with much larger R_h_ are observed. The complexes between Lys and HC 2 remained relatively stable for at least three months. Selected time-resolved DLS measurements are analyzed in the Supplementary Materials section (Figure S3 and Table S2).

**Figure 7:**
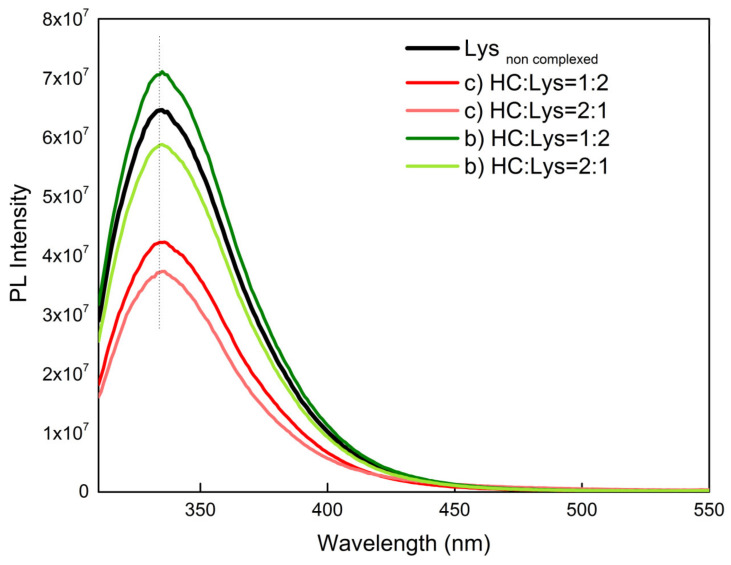
Fluorescence spectra of selected HC–Lys complexes.

Supplementary fluorescence experiments were performed for selected samples in order to investigate whether Lys preserves its spectral characteristics after complexation, which are directly connected to possible protein conformational changes that in turn may affect its bioactivity. Usually, denaturation signs are reflected in alterations of the characteristic fluorescence spectra of tryptophan residues [47,50]. As shown in Figure 7, the curve of the emission spectra, as well as the position of the emission maximum (λ_max_ = 335 nm) are identical for non-complexed and complexed Lys, displaying only intensity variations due to varying concentrations of Lys in the solutions. It is worth mentioning that the increase of fluorescence intensity is proportional to the concentration of Lys within the series of HC:Lys complexes, whereas this does not apply to comparisons of different Lys concentrations between complexes in cases (b) and (c). For example, comparing samples (c) HC:Lys = 1:2 and (b) HC:Lys = 1:2, the complex of series c with a higher concentration of Lys in the solution displays lower fluorescence intensity, suggesting that some tryptophan residues may be located in less water exposed areas or that quenching phenomena are taking place [51]. Overall, there are no signs of structural reorganization of Lys after complexation with the hyperbranched polyelectrolyte aggregates.

### 3.4. Curcumin Drug Loading Studies

**Figure 8:**
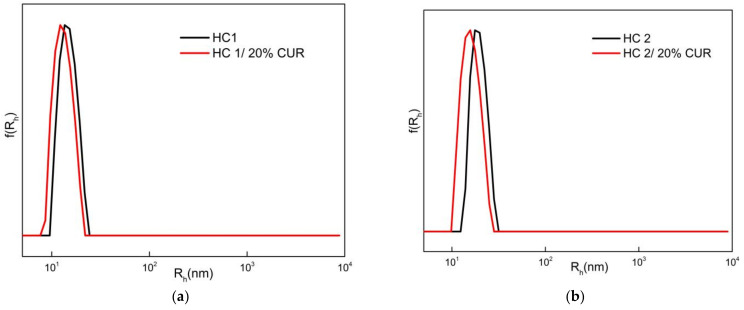
Size distributions from DLS analysis for the neat and CUR loaded NPs of HC 1 (**a**) and HC 2 (**b**).

In order to investigate the HCs drug encapsulation ability, the hydrophobic drug curcumin (CUR) was utilized. CUR is a bioactive substance known for its therapeutical anti-tumor and anti-inflammatory action as well as its fluorescent properties. Due to CUR very low solubility in water (approximately 11 ng/mL) [52], it is commonly employed as a model compound to explore the capacity of amphiphilic NPs to host drugs or imaging modalities in their hydrophobic core [53,54,55]. Based on a protocol described in the Methods section, CUR-loaded PNPs of 20% *w*/*w* targeted encapsulation of CUR were prepared. Both HC 1 and HC 2 generated transparent colored dispersions, representative of the yellowish tint of CUR, showing, at least from a qualitative perspective, no signs of CUR precipitation. A photograph of the HC/CUR mixed solutions is provided in Figure S5. Characterization of the CUR-loaded PNPs in terms of size and homogeneity was performed by DLS measurements. DLS histograms shown in Figure 8 and I, R_h_ as well as PDI values presented in Table 4, display a comprehensive comparison between CUR-loaded and neat HC NPs. Regarding both systems of HC 1 and HC 2, slightly decreased R_h_ and strongly decreased scattered light intensity values are observed. Hence incorporation of CUR in the polymeric nanosystems provokes different self-assembly that leads to NPs of smaller size and mass. Possibly, the increase in hydrophobic interactions renders the systems unable to withhold or even form aggregates consisting of a large number of hyperbranched chains. As for the effect of chemical composition in the CUR-loaded assemblies, HC 1 seems to form smaller and denser (higher I and lower R_h_ values) NPs compared to HC 2. 

CUR loaded PNPs were also characterized by means of zeta potential through ELS. Zeta potential for HC 1 decreases when CUR is present in the nano system, whereas contrarily, for HC 2 the value increases, in a way that even CUR-loaded NPs of HC 1 containing lesser MAA units display a more negative value. The number of polymer chains participating in the aggregates and their arrangement may be a contributing factor to this behavior.

UV-Vis spectroscopy was next employed in order to confirm the effective encapsulation of CUR in the PNPs. CUR presents different UV-Vis absorption spectra depending on the solution medium since as a 1,3-diketone moiety it exists in two possible forms, the enol-keto and keto-keto tautomer [56]. Due to CUR’s low water solubility, often mixed solutions of water and organic solvents are used as a standard to compare the photophysical properties of CUR-loaded NPs in water. In general, a maximum broad peak around 420 nm, a shoulder around 360, and a weak band around 260 nm are observed. Absorption at 350–420 nm is representative of π→π * transitions attributed to the feruloyl groups of CUR, whereas absorption at the lowest wavelength region of the spectrum ca. 260 nm is representative of forbidden n→π * transitions. The intensity and exact position of the peaks depend on a series of parameters, such as inter- or intramolecular hydrogen bonding, dipole–dipole interactions, and hydrophobic interactions. Absorption maximum around 420 (ranging from 408 to 430 in most organic solvents), which confirms the presence of enol-keto form, usually blue-shifts in non-polar and red-shifts in polar environments. In contrast, absorption around 360 confirms keto–keto form [57,58,59,60].

**Figure 9:**
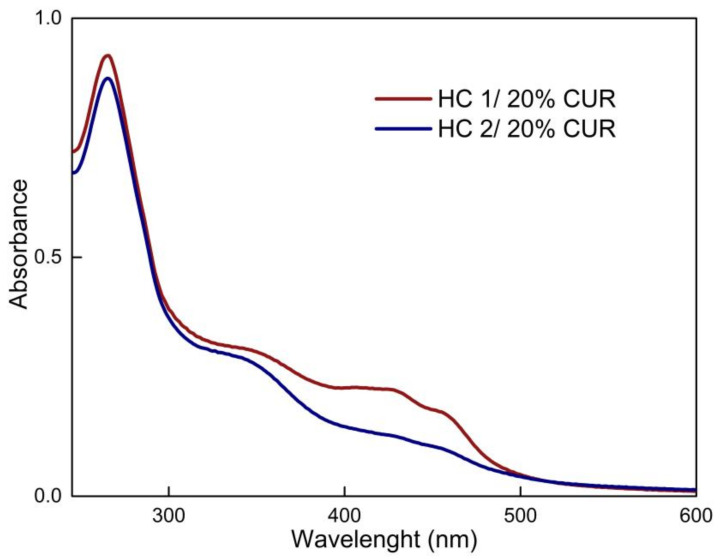
UV-Vis spectra of the CUR loaded PNPs.

UV-Vis spectra of HC 1/20% CUR and HC 2/20% CUR diluted samples are shown in Figure 9. A sharp peak at 265 nm, a broad band at 337 nm, and a broad band at 424 nm with small shoulders at 407 and 455 nm are observed for both HCs, while HC 1 spectra are of slightly higher intensity, especially in the 400–460 nm region. Apparently, both CUR tautomers exist in the solutions. It was reported that low intensity, around 420 nm, indicates non-polar, hydrophobic environment, and high intensity around the shoulder at 340 nm is a result of a more polar environment [57]. Therefore the spectra are somehow in agreement with the structure of the HCs, since HC 2 with more hydrophobic domains alongside with more polar PNP surface exhibit almost equal absorbance intensity to HC 1 around 340 nm, yet a much lower intensity is observed around 420 nm.

**Figure 10:**
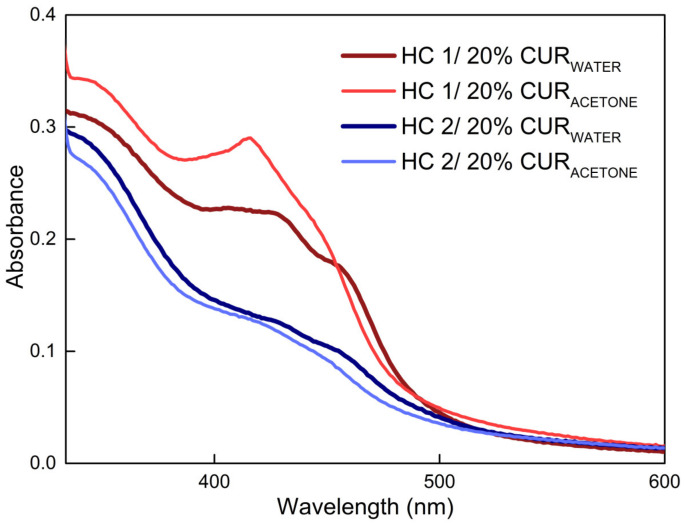
UV-Vis spectra of the CUR loaded PNPs upon dilution in acetone and water. (C_HC_ = 1.67 × 10^−4^ g/mL).

For a better inspection of the phenomena, analogous dilutions of the HC 1/20% CUR and HC 2/20% CUR samples in acetone were prepared. Figure 10 shows the superimposed UV-Vis spectra of CUR-loaded PNPs upon dilution in acetone and water. Dilution of the systems in acetone is expected to cause a collapse of the assemblies and, as a result, an increase of the absorbance intensity, since CUR is theoretically released from their hydrophobic domains and photochemical phenomena are more easily detected. CUR-loaded NPs of HC 1 display the described behavior upon dilution in acetone, where a sharper peak of higher intensity is observed at 417 nm and the shoulder at 450 nm does not appear, evidencing that CUR was indeed encapsulated in the PNPs and that intermolecular or intramolecular interaction took place. However, this is not observed for HC 2, where spectra remain almost identical. Since it is not possible for the assemblies to remain intact upon dilution in acetone, it is speculated that absorption of total CUR was already measured while the system was assembled, which is in accordance with the somewhat larger and looser PNPs studied by DLS measurements. CUR could be entrapped in residues of the hyperbranched surface of the PNPs where there is no hydrophobic protective environment; hence, some degradation may take place. From the UV-Vis spectra of the solutions in acetone, drug loading content and drug loading efficiency were calculated according to the equations described in the Supplementary Material (Table S3). CUR solubility was determined as 7 μg/mL for HC 1/20% CUR and 2 μg/mL for HC 2/20% CUR.

**Figure 11:**
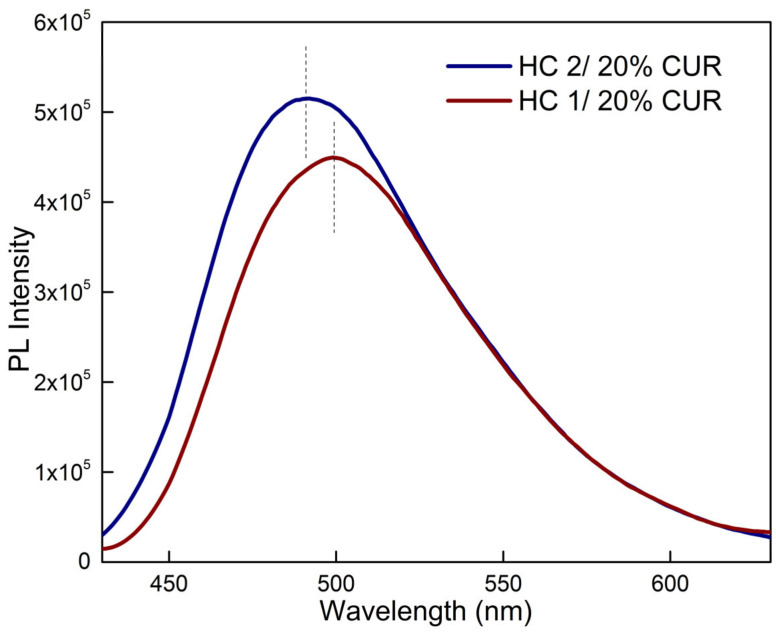
Fluorescence spectra of the CUR loaded PNPs.

Following the corroboration of CUR entrapment in the nano-systems through light scattering and UV-Vis spectroscopy experiments, the CUR-loaded PNPs were further studied in terms of their fluorescent properties. Fluorescence spectra of CUR are also sensitive to the polarity of the microenvironment around CUR. Free CUR in aqueous solution is reported to exhibit a very low broad peak at 540 nm, while in hydrophobic domains of micellar cores, a blue shift of the peak occurs [61,62]. HC 1/20% CUR and HC 2/20% CUR-diluted samples presented strong fluorescence as seen in Figure 11, with an emission maximum at 500 and 491 nm, respectively. Accordingly, HC 2, possessing a larger hydrophobic content, presents a greater blue shift. Unexpectedly, HC 2/20% CUR sample shows a more intense fluorescence signal, even though the calculated concentration of CUR is lower than that of HC 1/20% CUR. This may be attributed to self-quenching phenomena in the CUR-loaded HC 1 NPs, meaning that CUR is densely packed in these NP hydrophobic domains. Nonetheless, encapsulation of CUR in PNPs increases its solubility in the aqueous medium about 1000 times. 

The CUR loaded PNPs presented stability for a time period of approximately 15 days. After that period, both HC 1 and 2 systems showed a breakthrough where a high proportion of looser, much larger aggregates were formed (Figure S4 and Table S4). The corresponding bimodal distributions, analyzed by DLS, indicate that some of the initially formed assemblies tend to aggregate further. 

## 4. Conclusions

Hyperbranched P(OEGMA-co-tBMA-co-MAA) copolymers of unique macromolecular architecture and composition were synthesized via RAFT polymerization and post polymerization hydrolysis reaction. Polymerization reaction resulted in high conversion of relatively homogenous copolymers, while the hydrolysis reaction presented a small overall conversion depending on the copolymer composition. The copolymers, possessing amphiphilic and polyelectrolyte character, were studied by light scattering and spectroscopic techniques, in terms of their self-assembly behavior. Self-assembly properties were found to be composition and pH-dependent, while low CAC values were observed. Additionally, HC 2 NPs were capable of forming complexes with the cationic protein Lys through electrostatic interactions, whereas both HC 1 and 2 were able to incorporate the hydrophobic drug CUR when self-assembled in aqueous media. The complexed and loaded NPs systems studied were characteristic as to their small sizes and fluorescence properties, rendering the HCs as a candidate delivery system for bio-relevant compounds.

## Figures and Tables

**Table 1:** Molecular characteristics of the synthesized HCs.

HC	Initial Feed Ratio ^c^	M_w_^a^ (g/mol) ( ×10^4^)	M_w_/M_n_ ^a^	%wt tBMA ^b^ before Hydrolysis	%wt tBMA ^b^ after Hydrolysis
HC 1	[7.4]:[21.1]:[2]:[1]:[0.5]	1.5	1.29	34	32
HC 2	[5.3]:[35.2]:[2]:[1]:[0.5]	1.6	1.26	54	39

^a^ Determined by SEC in THF/5% Et_3_N, ^b^ Determined by ^1^H-NMR in CDCl_3_, ^c^ [OEGMA]:[tBMA]:[EGDMA]:[CTA] mole ratio.

**Table 2:** DLS results of HCs in aqueous media in varying pH solutions.

HC Samples	I_90°_ (a.u.)	R_h_ (nm)	PDI	ζ_p_ (mV)	CAC (g mL^−1^)	I_1_/I_3_^a^
HC 1 _pH3_	520	14	0.55	−1.6	--	--
HC 1 _pH7_	780	15	0.51	−5.5	2.6 × 10^−6^	1.2
HC 1 _pH10_	290	16	0.53	--	--	--
HC 2 _pH3_	500	29	0.38	−10.5	--	--
HC 2 _pH7_	1200	19	0.44	−32.8	2.2 × 10^−6^	1.2
HC 2 _pH10_	110	13	0.51	−24.9	--	--

^a^ Values for HC concentrations were aggregates are formed.

**Table 3:** DLS results for the three series of HC–Lys complexes.

Series of Complexes	HC:Lys Ratio	C_polymer_ (g/mL)	C_lysozyme_ (g/mL)	I_90°_ (a.u.)	PDI	R_h_ (nm)	ζ_p_ (mV)
a	HC _Non complexed_	0.43 × 10^−4^	-	48	0.44	19	−13.9
a	HC:Lys = 2:1	0.43 × 10^−4^	0.69 × 10^−4^	107	0.51	12 (47%)/99 (53%)	+2.3
a	HC:Lys = 1:1	0.43 × 10^−4^	1.38 × 10^−4^	67	0.47	25	+0.8
b	HC _Non complexed_	0.75 × 10^−4^	-	212	0.13	24	−18.9
b	HC:Lys = 2:1	0.75 × 10^−4^	1.19 × 10^−4^	235	0.16	22	+1.5
b	HC:Lys = 1.5:1	0.75 × 10^−4^	1.59 × 10^−4^	230	0.17	23	+1.0
b	HC:Lys = 1:1	0.75 × 10^−4^	2.39 × 10^−4^	225	0.17	24	+0.4
b	HC:Lys = 1:1.5	0.75 × 10^−4^	3.58 × 10^−4^	215	0.17	24	+1.4
b	HC:Lys = 1:2	0.75 × 10^−4^	4.77 × 10^−4^	235	0.17	24	+0.7
c	HC _Non complexed_	1.87 × 10^−4^	-	136	0.32	15	−16.4
c	HC:Lys = 2:1	1.87 × 10^−4^	2.97 × 10^−4^	365	0.27	25	+1.2
c	HC:Lys = 1.5:1	1.87 × 10^−4^	3.96 × 10^−4^	348	0.25	23	+6.4
c	HC:Lys = 1:1	1.87 × 10^−4^	5.93 × 10^−4^	275	0.27	22	+8.8
c	HC:Lys = 1:1.5	1.87 × 10^−4^	8.91 × 10^−4^	261	0.30	23	+0.7
c	HC:Lys = 1:2	1.87 × 10^−4^	11.87 × 10^−4^	276	0.30	23	+1.4

**Table 4:** Comparison of DLS and ELS results for the neat and CUR loaded PNPs.

HC Samples	I_90°_ (a.u.)	R_h_ (nm)	PDI	ζ_p_ (mV)
HC 1	780	15	0.51	−5.5
HC 1/20% CUR	380	13	0.55	−23.3
HC 2	1200	19	0.44	−32.8
HC 2/20% CUR	185	16	0.56	−14.8

## Data Availability

Data are available on request.

## Supplementary Materials

The following supporting information can be downloaded at: https://www.mdpi.com/article/10.3390/ma16031045/s1, Figure S1: FT-IR spectra of HC 1 before and after hydrolysis; Figure S2. Size distributions of HC 1 (a) and HC 2 (b) in THF solution at c = 10^−2^ g/mL obtained by DLS measurements; Table S1: DLS results for the HCs before and after hydrolysis in THF (c = 10^−2^g/mL); Figure S3: Size distributions from DLS measurements of sample HC: Lys = 1:1 (A) and HC: Lys = 1:2 (B) of series (c), between a time period of three months; Table S2: DLS analysis of sample HC: Lys = 1:1 and HC: Lys = 1:2 of series (c), between a time period of three months; Figure S4: Size distributions from DLS measurements of samples HC 1/20% CUR (a) and HC 2/20% CUR (b) between a time period of approximately five months; Figure S5: Photograph of the CUR mixed HCs in aqueous solutions; Table S3: DLE and DLC values based on UV-Vis absorption. Table S4: DLS analysis of samples HC 1/20% CUR (a) and HC 2/20% CUR (b) between a time period of approximately five months.
