# Supplementary material for: Hyperbranched Polyelectrolyte Copolymers as Novel Candidate Delivery Systems for Bio-Relevant Compounds
Source: Materials (Basel). 2023 Jan 24;16(3):1045. doi: 10.3390/ma16031045 (PMC9921860; doi:10.3390/ma16031045)
Supplement: Supplementary file 1 [file materials-16-01045-s001.zip › materials-2160281-supplementary.pdf]

materials

Supplementary Materials

Hyperbranched Polyelectrolyte Copolymers as Novel Candidate Delivery Systems for Bio-Relevant Compounds

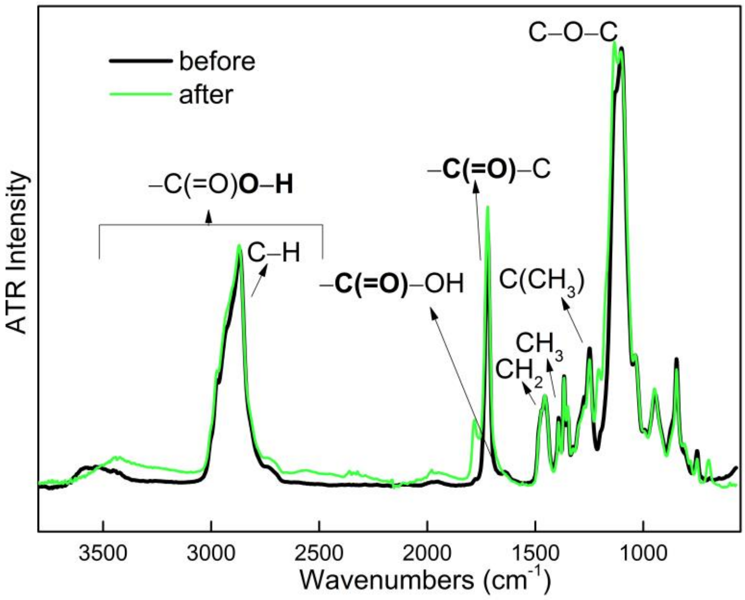

Figure S1. FT-IR spectra of HC 1 before and after hydrolysis.

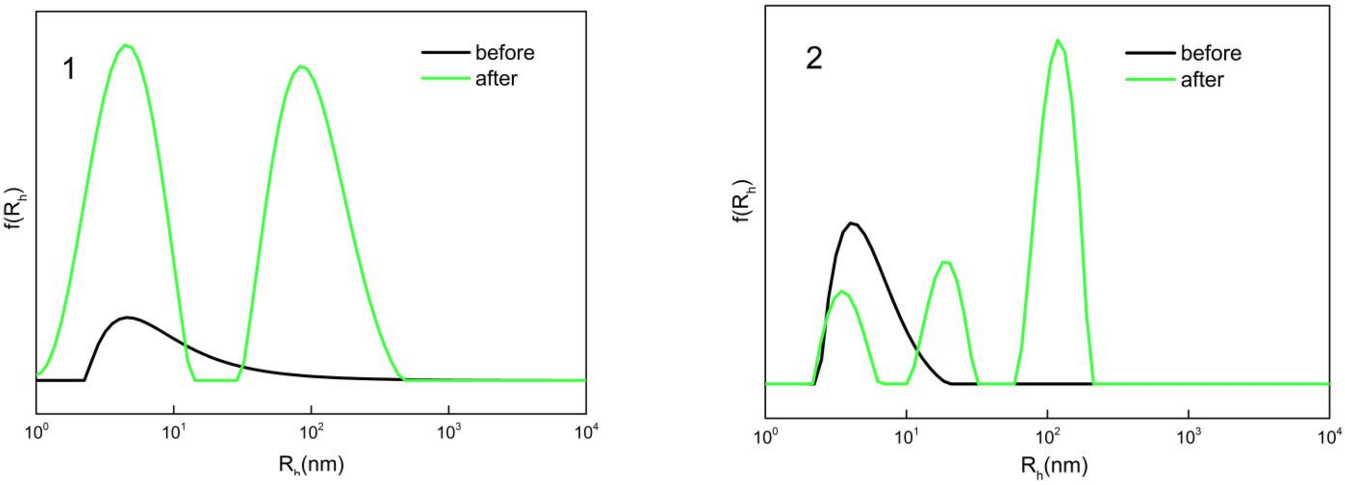

(**a**) (**b**)

Figure S2. Size distributions of HC 1 (**a**) and HC 2 (**b**) in THF solution, before and after hydrolysis, at c= 10^{-2}g/ml obtained by DLS.

Table S1. DLS results for the HCs before and after hydrolysis in THF (c= 10^{-2}g/ml).

HC	$I_{90°}$ (a.u.)	R_h(nm)	PDI
HC 1 before hydrolysis	60	9	0.54
HC 1 after hydrolysis	68	98 (46%)/ 3 (54%)	0.48
HC 2 before hydrolysis	80	5	0.57
HC 2 after hydrolysis	218	3 (15%)/ 18 (20%)/ 116 (63%)	0.53

Stability studies

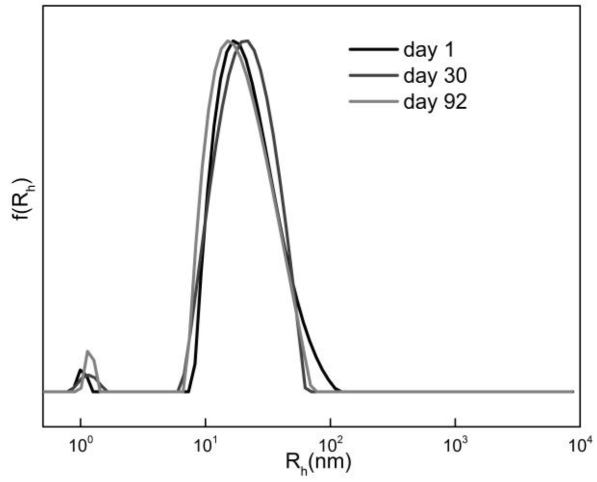

(**A**)

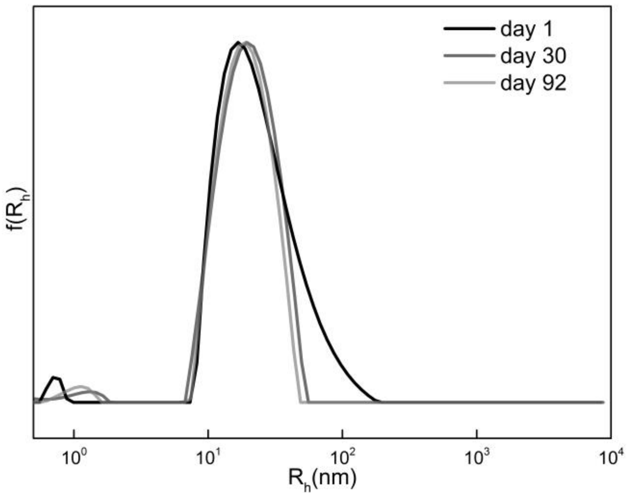

(**B**)

Figure S3. Size distributions from DLS measurements of sample HC: Lys =1:1 (A) and HC: Lys =1:2(B) of series (c), within a period of three months.

Table S2. DLS analysis of sample HC: Lys =1:1 and HC: Lys =1:2 of series (c), within a period of three months.

Sample	Time	$I_{90°}$ (a.u.)	R_h(nm)	PDI
(c) HC: Lys =1:2	Day 1	276	23	0.30
	Day 30	207	20	0.28
	Day 92	220	19	0.27
(c) HC: Lys =1:1	Day 1	275	22	0.27
	Day 30	252	21	0.27
	Day 92	237	19	0.28

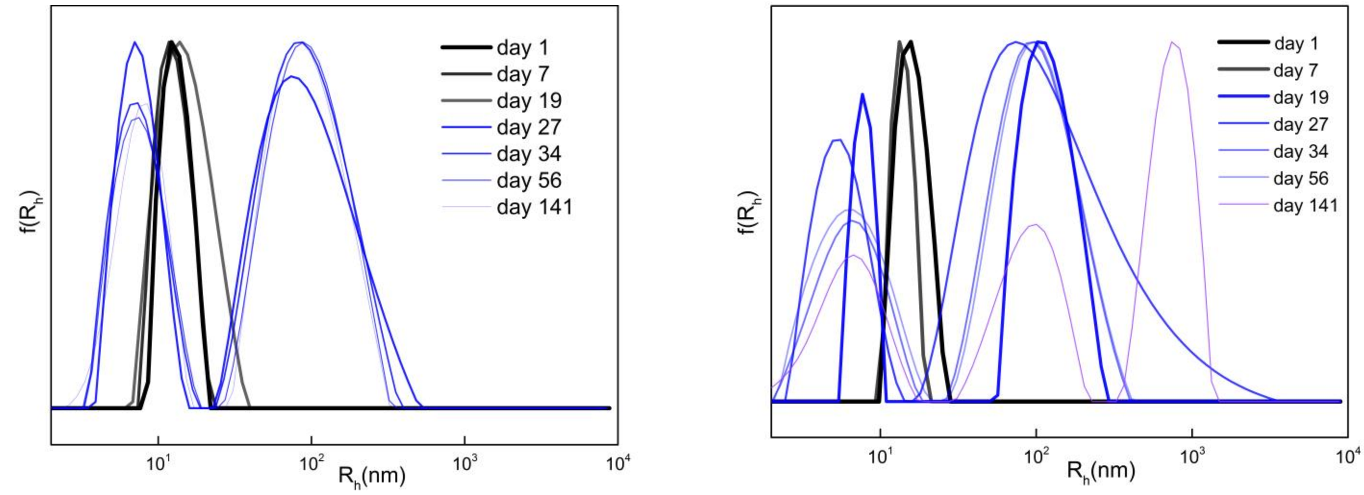

(**a**) (**b**)

Figure S4. Size distributions from DLS measurements of samples HC 1/20% CUR (a) and HC 2/ 20% CUR (b) within a time period of approximately five months.

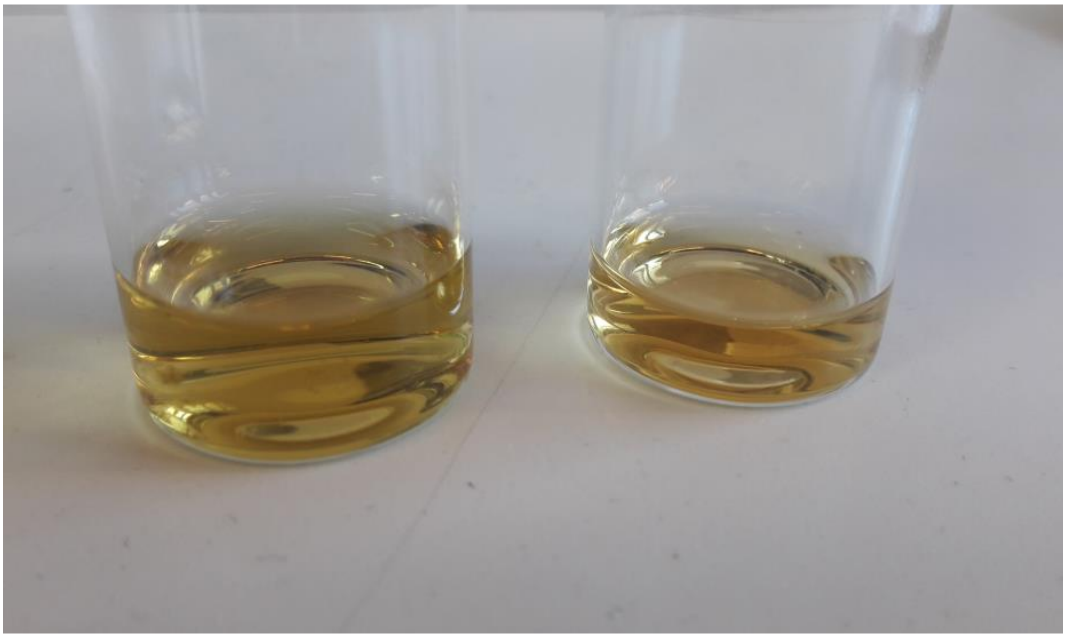

Figure S5. Photograph of the HC/CUR mixed NPs in aqueous solutions.

Table S3. DLE and DLC values based on UV-Vis absorption measurements.

**Sample**	**DLC (wt%)**	**DLE (wt%)**
HBC 1/CUR 20%	1.4	7.3
HBC 2/CUR 20%	0.4	2.2

Table S4. DLS analysis of samples HC 1/20% CUR (a) and HC 2/ 20% CUR (b) within a period of approximately five months.

Sample	Time	$I_{90°}$ (a.u.)	R_h(nm)	PDI
HC 1/20% CUR	Day 1	380	13	0.55
	Day 7	355	12	0.54
	Day 19	316	15	0.54
	Day 27	310	92(66%)/ 7(34%)	0.51
	Day 34	315	91(66%)/ 7(34%)	0.53
	Day 56	290	93(65%)/ 8(35%)	0.52
	Day 141	330	91(65%)/ 8(35%)	0.52
HC 2/20% CUR	Day 1	185	16	0.56
	Day 7	180	14	0.55
	Day 19	175	117(71%)/ 8(29%)	0.57
	Day 27	200	119(76%)/ 6(23%)	0.53
	Day 34	225	99(69%)/ 6(31%)	0.54
	Day 56	200	102(66%)/ 6(34%)	0.52
	Day 141	339	731(44%)/ 88(30%)/ 6(26%)	0.50

Drug loading content (DLC) and Drug loading efficiency (DLE)

DLC and DLE for the CUR loaded PNPs were calculated using the equations:

$$\text{DLC (wt\%)} = (\text{weight of loaded drug/ weight of drug-loaded polymer}) \times 100, \quad \text{(S1)}$$

$$\text{DLE (wt\%)} = (\text{weight of loaded drug/ weight of initial drug feed}) \times 100, \quad \text{(S2)}$$
